# Diversity of biting midges *Culicoides* (Diptera: Ceratopogonidae), potential vectors of disease, in different environments in an Amazonian rural settlement, Brazil

**DOI:** 10.1590/0037-8682-0067-2020

**Published:** 2020-05-18

**Authors:** Emanuelle de Sousa Farias, Jessica Feijó Almeida, Jordam William Pereira-Silva, Luiz de Souza Coelho, Claudia María Ríos-Velásquez, Sérgio Luiz Bessa Luz, Felipe Arley Costa Pessoa

**Affiliations:** 1 Fundação Oswaldo Cruz, Instituto Leônidas e Maria Deane, Laboratório de Ecologia e Doenças Transmissíveis na Amazônia, Manaus, AM, Brasil.; 2 Fundação Oswaldo Cruz, Instituto Oswaldo Cruz, Programa de Pós-Graduação em Biodiversidade e Saúde, Rio de Janeiro, RJ, Brasil.; 3 Instituto Nacional de Pesquisas da Amazônia, Programa de Pós-Graduação em Entomologia, Manaus, AM, Brasil.; 4 Universidade do Estado do Amazonas, Escola Superior de Ciências da Saúde, Programa de Pós-Graduação em Medicina Tropical, Manaus, AM, Brasil.; 5 Fundação de Medicina Tropical Dr. Heitor Vieira Dourado, Departamento de Ensino e Pesquisa, Manaus, AM, Brasil.; 6 Instituto Nacional de Pesquisas da Amazônia, Laboratório de Inventário Florístico e Botânica Econômica, Coordenação de Biodiversidade, Manaus, AM, Brasil.

**Keywords:** Culicoides, Diversity, Abundance, Anthropized environments

## Abstract

**INTRODUCTION:**

The *Culicoides* transmit a variety of pathogens. Our aim was to survey the *Culicoides* species occurring in an Amazonian rural settlement, comparing abundance, richness, and diversity in different environments.

**METHODS:**

*Culicoides* were captured using CDC light traps. The Shannon-Wiener (H’) and Rényi indices were used to compare species diversity and evenness between environments, the equitability (J’) index was used to calculate the uniformity of distribution among species, and similarity was estimated using the Jaccard similarity index. A permutational multivariate analysis of variance was applied to assess the influence of environment on species composition. A non-metric dimensional scale was used to represent the diversity profiles of each environment in a multidimensional space.

**RESULTS:**

6.078 *Culicoides* were captured, representing 84 species (45 valid species/39 morphotypes). H’ values showed the following gradient: forest > capoeira > peridomicile > forest edge. The equitability J’ was greater in capoeira and forests compared to peridomiciles and the forest edge. The population compositions of each environment differed statistically, but rarefaction estimates indicate that environments of the same type possessed similar levels of richness. Species of medical and veterinary importance were found primarily in peridomiciles: *C. paraensis,* vector of Oropouche virus; *C. insignis* and *C. pusillus*, vectors of Bluetongue virus; *C. filariferus, C. flavivenula, C. foxi,* and *C. ignacioi,* found carrying *Leishmania* DNA.

**CONCLUSIONS:**

This study indicates that diversity was higher in natural environments than in anthropized environments, while abundance and richness were highest in the most anthropized environment. These findings suggest that strictly wild *Culicoides* can adapt to anthropized environments.

## INTRODUCTION

Tropical forest ecosystems host two thirds of the Earth’s terrestrial biodiversity and provide significant benefits to the biosphere and the global economy^1^. According to Steege et al. (2015)^2^, approximately 40% of the original Amazon forest will be lost by 2050 if historical rates of deforestation continue. Anthropization is the primary cause of environmental change and the degradation of tropical ecosystems.

Environmental changes caused by anthropogenic interference are associated with increased health risks. Vectors and pathogens previously found only in forests have been observed in human settlements. According to Gottdenker et al. (2014)^3^, disease transmission is affected land-use changes that include: deforestation, forest and habitat fragmentation, agricultural development, irrigation, urbanization, and suburbanization. In the Amazon, these changes may be responsible for the increased incidence of endemic diseases such as Malaria, Yellow fever, Dengue, Zika, Mayaro, Oropouche, Chikungunya, and several other arboviruses and protozoa that cause leishmaniasis.

Hematophagous biting midge populations are affected by anthropogenic interference. In the Brazilian Amazon, Castellón (1990)^4^ observed a greater abundance of biting midges in capoeira and clearings than in primary forest. In urban and rural areas in Maranhão State, Silva and Carvalho (2013)^5^ found both a greater richness and abundance of biting midges in peridomiciles. According to Cazorla and Campos (2018)^6^, anthropization impacts ceratopogonid communities by decreasing biodiversity and favoring species that best adapt to altered environments. 

This study was conducted in the settlement of Rio Pardo, Presidente Figueiredo Municipality, Amazonas State, Brazil. Until recently, human activity in Rio Pardo was limited to subsistence farming, raising livestock, hunting, fishing, and gathering wood for local use, but newly expanded branches of forestry and fish farming have had a significant impact on the natural environment. Disease transmission dynamics in the region may have been altered by the degradation of natural habitats causing increased human exposure to forests, and to disease vectors and their hosts. The increased supply of blood meal sources around peridomiciles and a lack of basic sanitation offer favorable conditions for insect vectors to flourish.

The genus *Culicoides* is comprised of 1.368 species worldwide; 299 of these species occur in the Neotropics and 122 species occur in the Brazilian Amazon Basin^7^ ^-^ ^8^. Certain *Culicoides* species transmit viruses such as African horse sickness virus (AHSV), Bluetongue virus (BTV) which infects domestic and wild ruminants, and Oropouche virus (OROV) which infect humans^9^. Oropouche virus is one of the most common arboviruses in Brazil; it has affected an estimated 500,000 people since it was first isolated in 1955^10^. Biting midges and simulids have been implicated in the transmission of some species of filariae to humans, including: *Mansonella ozzardi, M. perstans* and *M. streptocerca* ^11^. *Culicoides* may also be involved in the transmission of *Leishmania* ^12^.

Identifying possible disease vectors is therefore of significant epidemiological importance. The aim of this study was to survey the *Culicoides* species that occur in a typical Amazonian rural settlement, and to compare the abundance, richness, and diversity of *Culicoides* populations present in different environments.

## METHODS

**FIGURE 1:**
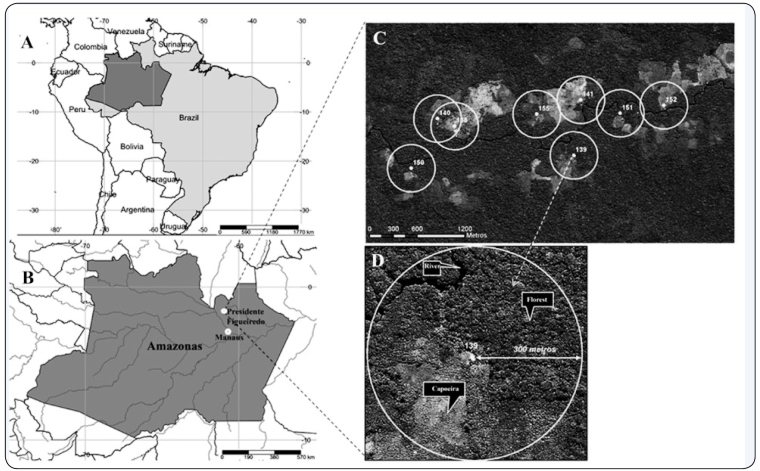
Location of the study area where *Culicoides* were captured: **(A)** Brazil; **(B)** Location of Presidente Figueiredo Municipality in the Central Amazon; **(C)** The rural settlement of Rio Pardo, numbers indicate specific dwellings where collections were made; **(D)** Diagram of a single collection area, a collection area was defined as the area within 300 m of a dwelling (Adapted by Ramos et al. 2015^13^).

The rural settlement of Rio Pardo (1°49’02.3’’S 60°19’03.5’’W), Presidente Figueiredo Municipality, Amazonas State, Brazil (Figure 1), was founded in 1996 and has approximately 700 inhabitants (ILMD/FIOCRUZ). In 2002, approximately 95% of its total area, about 28,000 hectares, was composed of preserved forest. From 1996-2002, the rate of deforestation was estimated to be about 150 ha/year, while land was developed for agricultural and community use at a rate of about 220 ha/year^13^.

*Culicoides* capture was performed in June, July, and August 2010 on four consecutive nights per month. Four different environments within the household buffer zone were sampled: peridomicile, areas with enclosures for livestock; capoeira, successional areas that have regenerated naturally from both functioning and abandoned agroecosystems; forest edge, areas of transition between capoeira and forest; and forest, comprised primarily of ombrophilous forest or “terra firme” (upland Amazonian forest that never floods) and river margins. Twenty-four household buffers were sampled. Four Centers for Disease Control (CDC) light traps per household were used to capture *Culicoides*, totaling 96 traps. These were installed 1.5 m above the ground and remained in the field from 6:00 p.m. to 6:00 a.m. 

Biting midges were transported in 70% ethanol and slide-mounted in phenol-balsam, as described by Wirth and Marston (1968)^14^. Species identification was performed following: Wirth and Blanton (1959)^15^, Spinelli et al. (1993)^16^, and Santarém et al. (2015)^17^; subgeneric classifications were based on Borkent (2015)^18^. Voucher specimens were deposited at the Laboratório de Doenças Transmissíveis na Amazônia (ILMD/FIOCRUZ, Amazônia). 

Many specimens had morphological variations in common. Unidentified specimens were therefore grouped into morphotypes and treated as valid species in the diversity and richness analyses. The Shannon-Wiener (H’) and Rényi indexes were used to compare species diversity and evenness between the four environments. The Pielou equitability index (J') was used to calculate the uniformity of distribution of individuals among species, and similarity was calculated using the Jaccard similarity index (Cj), which qualitatively compares species similarity along an environmental gradient^19^. A permutational multivariate analysis of variance (PERMANOVA) was applied to assess the influence of each environment on species composition. A non-metric dimensional scale (NMDS) was used to represent the diversity profiles of each environment in a multidimensional space. All analyses were carried using the statistical package R version 3.4.2^20^. The level of significance considered for all tests was 95%.

## RESULTS

A total of 6.078 *Culicoides* (96.38% females and 3.62% males) were collected, representing 84 species, comprised of 45 valid species and 39 morphotypes. 

*Culicoides fusipalpis* exhibited the highest abundance (2.178-35.84%), followed by *C. dasyophrus* (919-15.12%), *C. pseudodiabolicus* (459-7.55%), *C. diabolicus* (426-7.01%), *C. filarifer* (319-5.25%), *C. ocumarensi* (290-4.77%), *C. foxi* (231-3.80%), and *C. insignis* (229-3.77%). The remaining species represented less than 3% of the collected specimens (Table 1). The most abundant species in peridomiciles were *C. fusipalpis* (1.641-44.05%) and *C. diabolicus* (416-11.17%); the most abundant species in capoeira were *C. pseudodiabolicus* (27-31.77%) and *C. fusipalpis* (20-23.53%); the most abundant species at the forest edge were *C. dasyophrus* (745-47.79%) and *C. fusipalpis* (430-27.59%); and the most abundant species in forests were *C. pseudiabolicus* (246-34.75%) and *C. fusipalpis* (87-12.29%) (Table 1). 

 Of the incriminated and putative vectors recorded (Table 1): *C. paraensis* was found primarily in peridomiciles; *C. insignis* was most abundant in peridomiciles near corrals and chicken coops; a single specimen of *C. pusillus* was collected in a peridomicile near a corral; *C. filariferus* was found in all environments except capoeira; *C. flavivenula* exhibited low abundance in forests and peridomiciles; *C. foxi* occurred in all environments; and *C. ignacioi* occurred in all environments except forests.

Richness between environments varied from 22 to 58 species/morphotypes. Abundance varied significantly between environments, with values ranging from 85 to 3.725 individuals (Table 1). Species diversity was highest in forests (H’= 2.55) and lowest at the forest edge (H’=1.71). H’ values showed the following gradient: forest > capoeira > peridomicile > forest edge. The equitability (J’) index was greater in capoeira and forest than in peridomicile and the forest edge (Table 1), which indicates that *Culicoides* individuals are more equitably distributed among different species in capoeira and forest environments.

**TABLE 1:** *Culicoides* species collected in forests and anthropized environments in the rural settlement of Rio Pardo, Municipality of Presidente Figueiredo, Amazonas, Brazil.

	Environment				Total	%
Species	fo	fe	ca	pe		
*C. aldomani*	1	0	0	0	1	0,016
*C. baniwa*	0	0	0	3	3	0,049
*C. batesi*	2	0	0	8	10	0,165
C. benarrochi*	0	0	1	0	1	0,016
*C. bricenoi*	0	1	0	0	1	0,016
*C. brownie*	0	0	0	1	1	0,016
*C. castelloni*	1	0	0	0	1	0,016
*C. coutinhoi*	31	19	2	29	81	1,333
*C. dasyophrus*	7	745	4	163	919	15,123
C. debilipalpis*	17	10	0	2	29	0,477
*C. diabolicus*	2	7	1	416	426	7,010
*C. efferus*	0	0	0	4	4	0,066
*C. euplepharus*	0	0	0	5	5	0,082
*C. filarifer*	13	1	0	305	319	5,249
C. flavivenula*	1	0	0	2	3	0,049
*C. fluvialis*	0	1	0	0	1	0,016
C. fluviatilis*	0	0	0	1	1	0,016
C. foxi*	12	8	3	208	231	3,801
*C. franklini*	3	2	0	1	6	0,099
C. fusipalpis*	87	430	20	1641	2178	35,84
C. glabellus*	7	2	4	0	13	0,214
*C. glabrior*	3	1	1	9	14	0,230
*C. guamai*	1	1	0	0	2	0,033
*C. hylas*	54	30	3	26	113	1,859
C. ignacioi*	0	6	1	67	74	1,218
C. insignis*	6	6	0	217	229	3,768
*C. irreguralis*	0	0	0	1	1	0,016
C. leopoldoi*	18	66	1	10	95	1,563
C. limai*	67	6	0	5	78	1,284
C. lutzi*	3	3	0	14	20	0,329
*C. ocumarensi*	5	0	1	284	290	4,772
C. paraensis*	1	0	0	6	7	0,115
C. paraignacioi*	8	14	4	108	134	2,205
C. paramaruim*	0	0	0	1	1	0,016
*C. plaumanni*	26	21	3	0	50	0,823
*C. profundus*	11	7	0	1	19	0,313
C. pseudodiabolicus*	246	100	27	86	459	7,553
*C. pseudoreticulatus*	1	1	1	2	5	0,082
*C. pusilloides*	0	0	0	3	3	0,049
C. pusillus*	0	0	0	1	1	0,016
*C. rhombus*	1	1	1	1	4	0,066
*C. spurius*	1	0	0	0	1	0,016
*C. tetrathyris*	2	1	1	1	5	0,082
*C. tidwelli*	0	8	0	0	8	0,132
*C. verecundus*	18	1	1	7	27	0,444
*Hoffmania guttatus* sp. 23	0	0	0	1	1	0,016
*Ho. guttatus* sp. 24	0	0	0	3	3	0,049
*Ho. guttatus* sp. 25	0	0	0	1	1	0,016
*Ho. guttatus* sp. 26	0	0	1	0	1	0,016
*Ho. guttatus* sp. 27	4	0	0	0	4	0,066
*Ho. guttatus* sp. 28	0	1	0	1	2	0,033
*Ho. guttatus* sp. 29	0	2	0	0	2	0,033
*Ho. guttatus* sp. 30	0	0	0	4	4	0,066
*Ho. guttatus* sp. 31	1	0	0	0	1	0,016
*Ho. guttatus* sp. 32	1	0	0	0	1	0,016
*Ho. guttatus* sp. 33	0	0	0	2	2	0,033
*Ho. guttatus* sp. 34	13	0	0	0	13	0,214
*Ho. guttatus* sp. 35	0	0	0	5	5	0,082
*Ho. guttatus* sp. 36	1	0	0	2	3	0,049
*Ho. guttatus* sp. 37	4	13	1	8	26	0,428
*Ho. guttatus* sp. 38	0	0	0	27	27	0,444
*Ho. guttatus* sp. 39	10	7	3	2	22	0,362
*Ho. hylas* sp. 22	1	0	0	0	1	0,016
*Haematomyidium* sp. 1	0	1	0	3	4	0,066
*Ha.* sp. 2	0	23	0	11	34	0,559
*Ha.* sp. 3	0	3	0	2	5	0,082
*Ha.* sp. 4	0	0	0	1	1	0,016
*Ha.* sp. 5	1	0	0	0	1	0,016
*Ha.* sp. 6	4	0	0	0	4	0,066
*Ha.* sp. 7	0	0	0	1	1	0,016
*Ha.* sp. 8	1	0	0	0	1	0,016
*Ha.* sp. 9	1	0	0	0	1	0,016
*Ha. paraensis* sp. 13	0	9	0	0	9	0,148
*Ha. paraensis* sp. 14	0	0	0	2	2	0,033
*Ha. paraensis* sp. 15	0	0	0	1	1	0,016
*Ha. paraensis* sp. 16	0	0	0	1	1	0,016
*Ha. paraensis* sp. 21	0	0	0	3	3	0,049
Mataemyia sp. 17	0	0	0	2	2	0,033
group *limai* sp. 10	1	0	0	0	1	0,016
group *limai* sp. 11	7	0	0	0	7	0,115
group *limai* sp. 12	2	0	0	0	2	0,033
group *reticulatus* sp. 18	0	0	0	2	2	0,033
group *reticulatus* sp. 19	0	0	0	1	1	0,016
group *reticulatus* sp. 20	0	1	0	0	1	0,016
Abundance	708	1559	85	3725	6078	
Richness	46	37	22	58		
H’	2.55	1.71	2.32	2.11		
J’	0,66	0,47	0,75	0,51		

The abundance, richness, diversity indexes (H') and equitability (J') calculated for the *Culicoides* species collected in environments of forest (fo), forest edge (fe), capoeira (ca) and peridomicile (pe). *species known to exhibit anthropophilic behavior.

**FIGURE 2:**
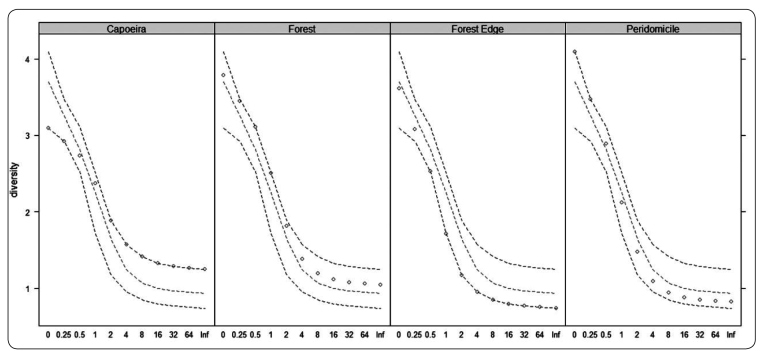
Rényi diversity profiles of *Culicoides* for the four environments investigated: capoeira (successional vegetation), forest, forest edge, and peridomicile in the rural settlement of Rio Pardo, Presidente Figueiredo Municipality, Amazonas, Brazil. Circles show the values for each site, and dashed lines show the median and extreme values.

**FIGURE 3:**
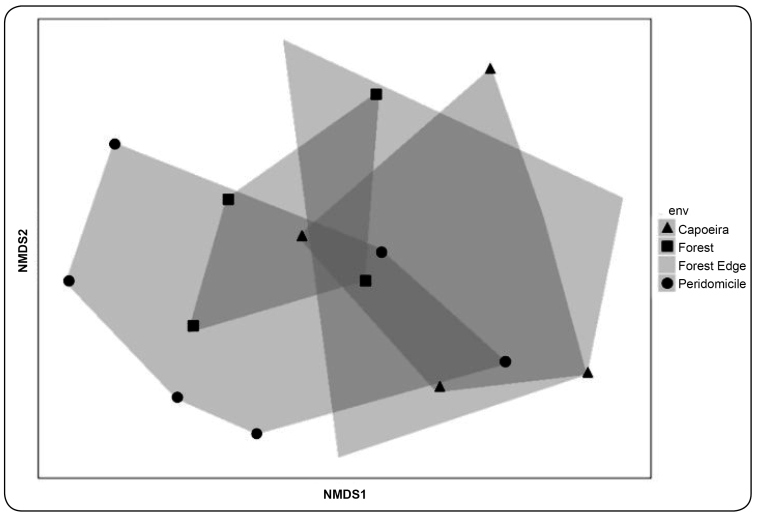
Non-metric multidimensional scaling (NMDS) showing *Culicoides* diversity profiles in capoeira, forest, forest edge, and peridomicile environments in the rural settlement of Rio Pardo, Presidente Figueiredo Municipality, Amazonas, Brazil.

**FIGURE 4:**
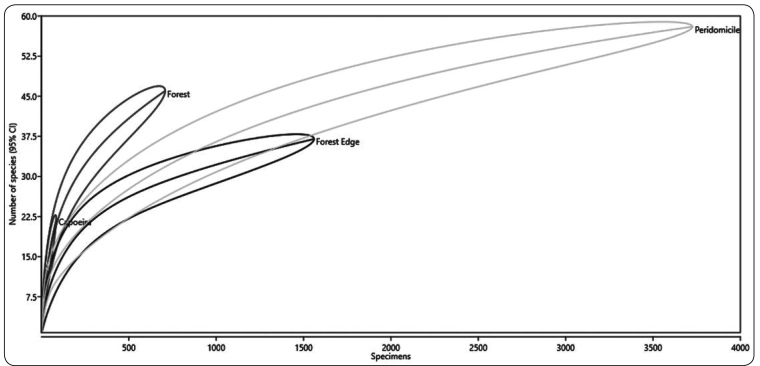
Rarefaction curves representing the species richness of *Culicoides* in capoeira, forest, forest edge, and peridomicile environments in the rural settlement of Rio Pardo, Presidente Figueiredo Municipality, Amazonas, Brazil (95% CI).

Similarity was greater between the forest and capoeira than between capoeira and peridomiciles. The population compositions of each environment differed statistically (PERMANOVA S.S=1.61, PSEUDO-F=1.96, p=0.04) (Figure 2 and Figure 3). Richness differed between environments, but rarefaction estimates indicate that environments of the same type possessed similar levels of richness (Table 1 and Figure 4). 

## DISCUSSION

Eighty-four *Culicoides* species/morphotypes were collected in the rural settlement of Rio Pardo, representing 69% of the known *Culicoides* species found in the Brazilian Amazon Basin^8^. Previous studies conducted in Rio Pardo recorded five new species of *Culicoides* and nine new occurrences^17^ ^,^ ^21^ ^-^ ^22^. Until now, the highest levels of richness in Brazil have been recorded in the municipality of Belém in Pará State, with 50 species; followed by Porto Velho in Rondônia, with 40 species; Alto Alegre in Roraima, with 38 species; and Manaus and Tefé in Amazonas, with 35 species and 19 species, respectively^23^ ^-^ ^25^.

Species of medical and veterinary importance were found primarily in peridomiciles, these include: *Culicoides paraensis*, which is a vector of OROV in humans; *C. insignis* and *C. pusillus*, which are vectors of BTV in domestic and wild ruminants; *C. filariferus* and *C. flavivenula*, which have been found carrying *Leishmania amazonensis* DNA; and *C. foxi* and *C. ignacioi* which have been found carrying *Le. braziliensis* DNA^9^ ^,^ ^26^. All of these species were found in this study, but some in low abundance. This is probably due to the collection method. In entomological inventories, it is common for some species to appear with low frequency because bait type, capture effort, collection environment, and the presence of animals favors the capture of other species^27^. This low frequency may also be related to the fact that some species are diurnal while others are nocturnal^28^ ^-^ ^29^.

Few studies conducted in the Amazon have examined *Culicoides* anthropophily. Of the species identified in this study, the following are known to exhibit anthropophilic behavior: *Culicoides batesi*; *C. benarrochei*; *C. debilipalpis*; *C. flavivenula*; *C. fluviatilis*; *C. foxi*; *C. fusipalpis*; *C. glabellus*; *C. ignacioi*; *C. insignis*; *C. leopondoi*; *C. limai; C. lutzi; C. paraensis*; *C. paraignacioi*; *C. paramaruim*; *C. pseudodiabolicus* and *C. pusillus* ^4^ ^,^ ^30^ ^-^ ^32^ *.* Of these, all except *C. glabellus* were found in peridomiciles. Santiago-Alarcon et al. (2013)^33^ argue that humans may serve as blood meal sources for dominant *Culicoides* species in peridomicile environments.

Abundance was greatest in peridomiciles, followed by the forest edge, forest, and capoeira. *Culicoides fusipalpis* has been observed in high abundance in anthropic environments where it feeds on a variety of blood meal sources, including humans, other mammals and birds^34^. Species richness was greatest in peridomiciles, followed by forest, forest edge, and capoeira. The concentration of a variety of blood meal sources and the presence of suitable breeding sites may be attracting *Culicoides* to peridomiciles. Santiago-Alarcon et al. (2013)^33^ surveyed *Culicoides* feeding behavior in households near an urban forest in Germany where the dominant *Culicoides* species were known to be ornithophilous and found that blood ingested by these midges contained the mitochondrial DNA of mammals such as cows and humans. Generalist species are able to tolerate a broad set of environmental conditions and make use of a wide range of resources, which allows them to become both widespread and locally abundant (Brown, 1984)^35^.

In Rio Pardo, species diversity was highest in forests, while richness and abundance was highest in peridomiciles. In a study conducted in Rondônia, Carvalho et al. (2016)^36^ found that species diversity was higher in forests than in pasture. In a study conducted in rural and urban areas in Maranhão, Silva and Carvalho (2013)^5^ found that species diversity was highest in the Cerrado (savanna) and in gallery forests, while richness and abundance were highest in peridomiciles.

Equitability was highest in capoeira environments, where the number of individuals per species ranged from 1 to 27, and equitability was lowest in the forest edge environments, where the number of individuals per species ranged from 1 to 745 (**Table 1**). The high equitability observed in capoeira environments is the result of low abundance and a relatively homogeneous distribution of species. The low equitability observed in forest edge environments is likely due to an uneven distribution of species abundance caused by the predominance of *C. dasyophrus*, which comprised 47.76% of all individuals collected at the forest edge. These findings demonstrate that the local diversity index may decrease in environments where one species is highly dominant.

Species similarity between environments was low (> 50%), which is likely due to the different degrees of anthropic interference present in each environment. Capoeira-forest edge and forest-forest edge exhibited the highest similarity. These findings suggest that strictly wild *Culicoides* fauna tends to adapt to anthropized environments.

We observed that richness differed between environments, but rarefaction estimates indicate that environments of the same type possessed similar levels of richness. This is probably due to the three-month capture effort occurring during the dry season, a period that the abundance of general hematophagous dipteria are low^13^, which may have interfered with the results, indicating the need for more captures and in different seasons.

The data obtained in this study indicate that diversity was higher in natural environments (forest) than in anthropized environments (capoeira), while abundance and richness were both highest in the most anthropized environment (peridomiciles). It is likely that the concentration of a variety of food sources and the presence of suitable breeding grounds in peridomiciles favors the establishment of certain species.

In settled areas, the presence of domestic and wild animals provides vectors with a rich variety of food sources and this fosters their adaptation to new environments. This behavior may alter pathogen transmission dynamics and increase the risk of disease transmission by *Culicoides.*
